# Significant Decrease in the Prevalence of Anxiety and Depression after Hepatitis C Eradication

**DOI:** 10.3390/jcm11113044

**Published:** 2022-05-28

**Authors:** Justyna Slonka, Damian Piotrowski, Ewa Janczewska, Arkadiusz Pisula, Joanna Musialik, Jerzy Jaroszewicz

**Affiliations:** 1 Department of Infectious Diseases and Hepatology, Medical University of Silesia, 41-902 Bytom, Poland; dpiotrowski@sum.edu.pl; 2 Department of Basic Medical Sciences, Faculty of Health Sciences, Medical University of Silesia, 41-902 Bytom, Poland; ejanczewska@sum.edu.pl; 3 ID Clinic, 41-400 Mysłowice, Poland; arkpis@poczta.onet.pl; 4 Department of Nephrology, Transplantation and Internal Medicine, Medical University of Silesia, 40-055 Katowice, Poland; jmusialik@sum.edu.pl

**Keywords:** hepatitis C, depression, anxiety, GAD-7, PHQ-9, HQLQv.2, WHOQOL-BREF, HCV eradication, HRQoL

## Abstract

Chronic hepatitis C (CHC) is an ongoing epidemiological problem. The hepatitis C virus (HCV) may infect brain tissue, worsening mental health outcomes. The new era of highly effective oral Direct-Acting Agents (DAA) has brought a chance to eradicate the infection by 2030, however, screening campaigns are urgently needed as the majority of the infected are still undiagnosed. The aim of this study was to assess the prevalence of anxiety and depression among HCV patients, and the correlation with health-related quality of life (HRQoL) in the real-world setting, before and after DAA treatment. Data on anxiety, depression, and HRQoL, were collected by using self-reported questionnaires in a single center in Poland. The study group involved 90 respondents, 50% female, with a mean age of 43.8 years. HCV eradication decreased anxiety prevalence from 30.4% to 19.1% and depression from 35.2% to 18.2%. Significant improvement in 3 out of 4 of the WHOQOL-BREF (TheWorld Health Organization Quality of Life-BREF) domains and 8 out of 10 of the HQLQv.2 domains was obtained. Anxiety diminished the somatic domain scores by 3.5 (*p* < 0.0001), psychological by 2.3 (*p* = 0.0062), social by 1.75 (*p* = 0.0008), and environmental by 2.68 points (*p* = 0.0029). Depression diminished the somatic domain scores by 3.79 (*p* < 0.001), psychological by 2.23 (*p* < 0.001), social by 1.84 (*p* < 0.001), and environmental by 2.42 points (*p* = 0.004). In the Hepatitis Quality of Life Questionnaire version 2 (HQLQ v.2), the presence of depression and/or anxiety-impaired mental health, physical health, well-being, and vitality. These results indicate the need for an active search for HCV-infective people, especially among patients in psychiatric and psychological care.

## 1. Introduction

The hepatitis C virus (HCV) is present in the blood and other tissues of an infected person. Thus, any procedure or activity leading to tissue disruption could be a potential risk factor for infection. Based upon the Annual Epidemiological Report for 2019 Hepatitis C, injection drug use was one of the three most common routes of transmission, and the routes by percent are: injection drug use (60% acute, 59% chronic), followed by male-to-male sexual contacts, and nosocomial transmission [1]. Only 20–30% of acutely infected individuals have any clinical symptoms [2]. Others remain unaware for many years and develop serious liver-related complications, leading to liver cirrhosis and hepatocellular carcinoma. Besides the destructive effects on hepatic cells, the virus can cause a broad spectrum of extrahepatic symptoms [3].

The central nervous system is one of the non-hepatic locations where HCV replicates [4]. Disorders such as depression, anxiety and fatigue, “brain fog”, cognitive impairment, encephalopathy, and related others, may be present in up to 50% of patients, regardless of the severity of the disease process in the liver [5]. Moreover, moderate to severe suicide risk prior to the treatment, assessed using the Depression Anxiety Stress Scale (DASS), is present in 18% of individuals [6]. These numbers are much higher than for the general population. Based on European data from the international study ESEMeD (European Study of the Epidemiology of Mental Disorders), which included more than 21,000 interviews, the criteria for major depression were met by 21% of respondents in France, 17.9% in the Netherlands, 14.1% in Belgium, 10.6% in Spain, 9.9% in Germany, and 9.9% in Italy [7]. Polish data showed a much lower prevalence of major depression in the general population (3%), but the authors suggest that these numbers may be underestimated, as more than 50% of the respondents refused to complete the survey [8].

The pathogenetic mechanisms describing the effects of HCV on the central nervous system are still not well understood. The virus that replicates in microglia cells causes their stimulation and, consequently, the release of cytokines and chemokines, leading to neuronal apoptosis, resulting in neurocognitive changes and depression [9]. In addition to biological determinants, another important factor causing depression and anxiety is stigma, experienced by HCV-infected patients [10]. As many as 57% of them experience stigma associated with the disease, which translates into an increased prevalence of depression (28% in the non-stigmatized group, 65% in the stigmatized group) and anxiety (56% and 82%, respectively) [11]. Depression and anxiety caused by social distance and feelings of rejection may translate directly or indirectly into a worsened response to antiviral treatment and work productivity [12].

In the era of interferon-based treatment, the efficacy was poor and the treatment itself was associated with worsening the patients’ quality of life and caused many side effects that often led to discontinuation of the therapy [13]. Simply being aware of the diagnosis at that time could affect perceived symptoms and impair the quality of life [14]. New oral Direct-Acting Agents (DAA) regimens are highly effective and safe, bringing a change in the treatment paradigms. While chronic disease has become manageable, the aspect of good quality of life for HCV-infected patients were recognized and gained importance.

Many clinical trials showed an improvement in patient-reported outcomes after HCV eradication [15]. Patients with mental disorders are often excluded from the clinical trials. Data linking the assessment of mental disorders and quality of life in the clinical practice setting are still insufficient.

The aim of our study was to prospectively analyze the prevalence of depression and anxiety, and to assess health-related quality of life (HRQoL) in patients with HCV infection, prior to and at the end (EOT) of the DAA treatment. We also investigated the correlation between depression, anxiety, and quality of life, and we identified factors deteriorating HRQoL in the real-world setting.

## 2. Materials and Methods

### 2.1. Study Design

A descriptive, prospective, survey study was conducted in the large private medical center ID Clinic in Southern Poland, from October 2017 till the end of 2018. The study included all of the patients who registered for chronic hepatitis C treatment during this period in this medical center, agreed to participate and met the following DAA-treatment qualifying criteria: (1) age ≥ 18 years; (2) diagnosis of chronic hepatitis C; (3) presence of HCV RNA in serum or liver tissue; (4) evidence of liver fibrosis determined by Transient Elastography (TE, Fibroscan^®^) and quantified according to METAVIR staging system [16].

Patients actively addicted to alcohol and psychoactive substances, not knowing the Polish language and those with active mental disorders, such as mania and schizophrenia, were excluded from the study. Pre-existing, pharmacologically treated depression was not a criterion for exclusion. 

All questionnaires and patient surveys were patient self-administrated. The surveys were initially reviewed by a nurse for evidence of major depression and suicide risk. In such cases, it was suggested that the spoke with a physician. None of the respondents required psychological help or additional pharmacological intervention during the study period. The questionnaires were collected at two time points: before DAA treatment inclusion; and at the end of the treatment (EOT) of DAAs. The knowledge of achieving SVR alone can cause a positive psychological effect in patients and result in a sense of improved quality of life [17]. For this reason, the second surveys were collected before the patient was informed of the treatment outcome. A total of 103 patients completed anonymous survey questionnaires. Following the survey scoring recommendations [18], surveys were excluded if 20% or more of response data were missing from the assessment. When a single item for a domain was missing, a response was imputed using the mean of the rest of the domain responses. For more than two items missing, the whole domain was not calculated and excluded from analysis, which is outlined in the “missing data” columns in each table. Thirteen surveys were discarded due to more than 20% missing data. A total of 90 patients were finally included. 

Patients were asked about duration of HCV infection, stage of fibrosis, coinfections with other viruses, and any previous HCV treatment. Quality of life was assessed with use of generic (WHOQOL-BREF) and specific for hepatitis (HQLQv.2) questionnaire with integrated SF-36 survey.

The effect of particular treatment options were not considered, due to extremely disparate sample sizes among the treatment options. The proportions of specific therapies in this center were as follows: grazoprevir/elbasvir 74%; glecaprevir/pibrentasvir 17%; ledipasvir/sofosbuvir 6%; sofosbuvir/velpatasvir 2%.

### 2.2. Ethical Consideration

The study was reported to the Bioethics Committee of Medical University of Silesia and granted a waiver of informed consent from study participants due to its prospective, survey-based, and anonymous design (consent no KNW/0022/KB/257/17).

### 2.3. Measurements

The World Health Organization Quality of Life (WHOQOL-BREF) Questionnaire [19] and the licensed Hepatitis Quality of Life Questionnaire™ version 2 (HQLQv.2™) (QualityMetric™, Inc., Johnston, RI, USA) [20], both in validated Polish versions, were used. For the screening of depression and anxiety, the PHQ-9 (Patient Health Questionnaire) [21] and GAD-7 (Generalized Anxiety Disorder) [22] questionnaires were used.

### 2.4. Statistical Analysis

Statistica 13.3 TIBCO^®^ Software (Palo Alto, CA, USA) was used for the data analyses. Descriptive statistical methods were used. For additional statistical analysis and graphs, program R 3.4.0 was used. Results were presented for all of the subjects as mean (±standard deviation) or median (Q1–Q3), as appropriate, and adjusted for gender, degree of fibrosis, time since diagnosis, and prior treatment for hepatitis C. The correlations between the scores on the individual questionnaires were evaluated using Spearman’s rank correlation coefficients. A *p* value less than 0.05 was considered significant.

## 3. Results

We collected questionnaires from 90 respondents qualified for DAA treatment. All participants completed the treatment period, with SVR = 100%. A total of 50% were female. The mean age was 43.8 ± 12.6 (range: 24–71), the leading age group (37.8%) was between 31 and 40 years old.

More than one-third (38.4%) of the patients became aware of their infection recently: within the last 2 years. Another 22.5% knew about their infection for 3–4 years, while 25.8% were aware of their infection for longer than 10 years (Table 1). The distribution of genotypes was as follows: 1b = 75%; 1a = 3%; 1 = 2%; 3 = 16%; 4 = 4%.

### 3.1. Prevalence of Anxiety and Depression

**Figure 1:**
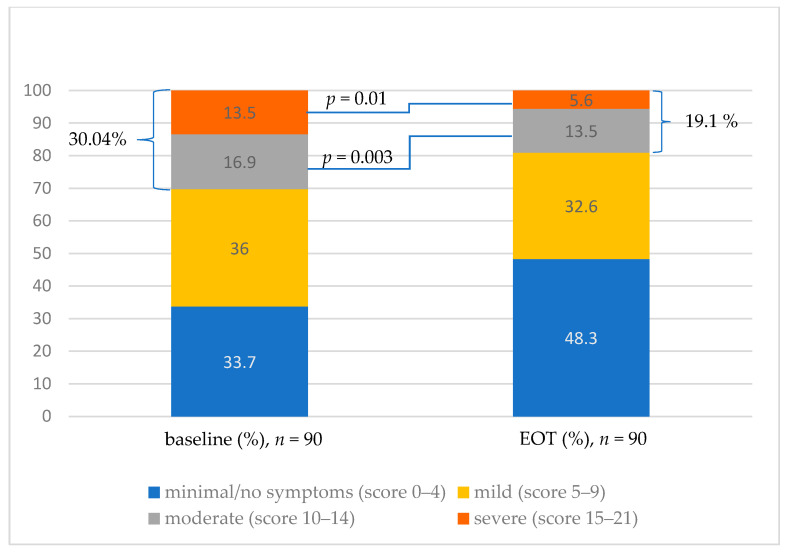
The results of anxiety classification according to the GAD-7 scale at baseline and at EOT. EOT—end of treatment.

A total of 30.4% of the HCV-infected patients presented symptoms of severe and moderate anxiety (cut-off > 10 point) at the baseline. In this group, almost half (13.5%) were those with severe anxiety (Figure 1). Seven patients (7.8%) had a history of depression and were treated with antidepressants at the time of the study. Unfortunately, it is not known how these particular patients performed on the screening test due to the blinding procedures.

After DAA treatment, the percentage of patients with a score > 10 points dropped to 19.1%, and the percentage of patients with severe anxiety dropped from 13.5% to 5.6% (*p* = 0.01). Almost half of the patients (48.3%) had no anxiety symptoms at EOT, with the *p* value approaching statistical significance (*p* = 0.055).

An extended follow-up was completed among the patients with pre-existing depressive disorder. Six months after the treatment, two of them were able to discontinue their antidepressants.

The mean pre- and post-treatment summed scores were 7.6 and 5.6, respectively. The post-treatment score was statistically lower (*p* < 0.001) with a difference of two points, which is a 26.3% reduction from the baseline (Table 2).

**Figure 2:**
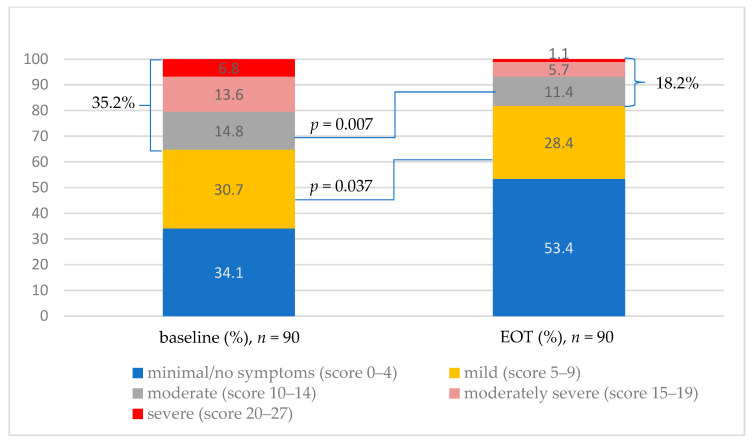
The results of depression classification according to the PHQ-9 scale at baseline and at EOT. EOT—end of treatment; PHQ-9—Patient Health Questionnaire.

The PHQ-9 questionnaire determined a significant degree of depression in the study population. As many as 35.2% of patients scored >10 points at the baseline. After DAA treatment, the proportion of those decreased almost twofold (18.2%). Moreover, only 1.1% of patients showed features of severe depression, compared to 6.8% before treatment, and the number of patients with moderately severe depression dropped by more than half (13.6% vs. 5.7%) (Figure 2)**.** Most patients with severe or moderately severe depression experienced clinical improvement after DAA treatment. However, the results were not statistically significant (*p* = 0.086). The decrease in mild and moderate depression was statistically important (*p* = 0.036, *p* = 0.007).

The percentage of patients with no depressive symptoms increased to more than half (53.4%) at EOT.

At the baseline, the mean summary score in the study group was 8.2 points and at EOT dropped to 5.5, which represents a 34.1% decrease (*p* < 0.001) (Table 2).

### 3.2. Anxiety and Depression Improvement in Subgroups

#### 3.2.1. Anxiety Subgroup Analysis

**Figure 3:**
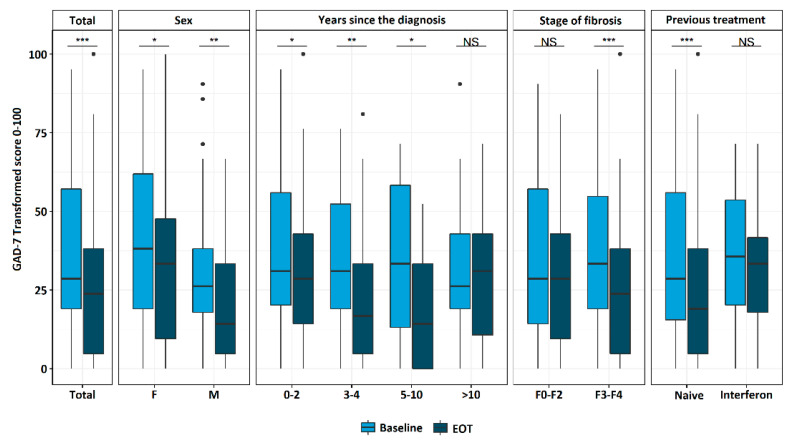
The scoring results of GAD-7 transformed to 0–100 scale at the baseline (light blue box) and at EOT (navy box) in the subgroups analysis. EOT—end of the treatment; GAD-7—Generalized Anxiety Disorder; F—female; M—male; * *p* < 0.05; ** *p* < 0.01; *** *p* < 0.001; NS—not significant.

After DAA treatment, a significant reduction of the scores in the GAD-7 questionnaire was observed in all of the subgroups except in the subgroup of patients who were ill for the longest period, and in those previously treated with interferon. The analysis showed that female respondents scored higher (worse) both before and after treatment. The results, transformed to a 0–100 scale, are shown in Figure 3.

#### 3.2.2. Depression Subgroup Analysis

**Figure 4:**
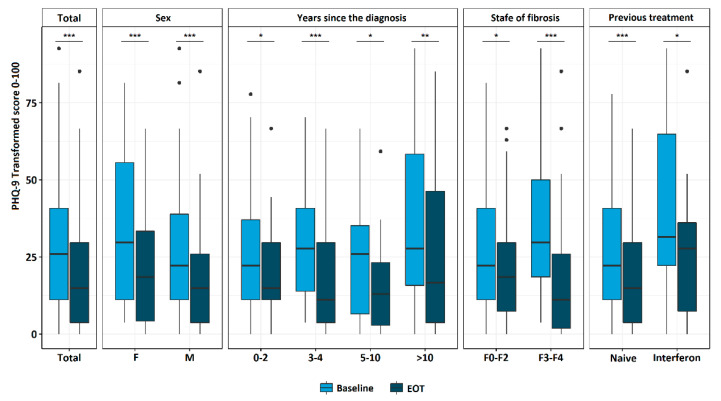
The scoring results of PHQ-9 transformed to 0–100 scale at the baseline (light blue box) and at EOT (navy box) in the subgroups analysis. EOT—end of the treatment; PHQ-9—Patient Health Questionnaire; F—female; M—male; * *p* < 0.05; ** *p* < 0.01; *** *p* < 0.001.

There was a significant improvement in the PHQ-9 questionnaire scores after DAA treatment in all of the studied subpopulations. The difference between the time points was most pronounced among patients with advanced fibrosis. In terms of depressive symptoms, female respondents also scored worse than male, at the baseline as well as after treatment. A statistically significant improvement after treatment was observed in both male and female respondents. The results, transformed to a 0–100 scale, are shown in Figure 4.

### 3.3. Quality of Life Improvement after HCV Eradication

#### 3.3.1. WHOQOL-BREF Results

The score represents the individual’s perception of quality of life in terms of four domains; if it has a positive direction, i.e., the higher the number of points obtained, the better the quality of life. The effect of treatment on each of the domains was analyzed, and the results are presented in Table 3. There was a statistically significant improvement after treatment in all of the domains except social. The most pronounced difference between baseline and final scores was noted in the somatic subscale.

#### 3.3.2. HQLQ v.2 Results

Significant differences were obtained in six of the eight subscales of the HQLQ v.2 after treatment, as well as in both of the disease-specific subscales “HCV distress” and “HCV limitations”.

There was a significant difference in the pre- and post-treatment scores of both PCS (Physical Component Score) and MCS (Mental Component Score) (Table 4). The difference in MCS was 4.47, which was an 8% change from the baseline score.

### 3.4. The Effects of Anxiety and Depression on Quality of Life Impairment

To assess the effect of anxiety and depression on quality of life reported in WHOQOL-BREF questionnaire, patients were divided into two groups, according to the GAD-7 and PHQ-9 scores. 

The result >10 points was considered as a presence of anxiety/depression, and <10 points as the absence of anxiety/depression.

Transitions from non-anxiety to anxiety resulted in worsening WHOQOL-BREF questionnaire scores in the somatic domain by 3.5 points, in the psychological domain by 2.3 points, in the social domain by 1.75 points, and in the environmental domain by 2.68 points. The results were statistically significant (Table 5).

Transition from non-depressed to depressed status resulted in a decrease in the WHO BREF questionnaire scores in the somatic domain by 3.79 points, in the psychological domain by 2.23 points, in the social domain by 1.84 points, and in the environmental domain by 2.42 points (Table 5).

#### Correlation between Questionnaires

**Figure 5:**
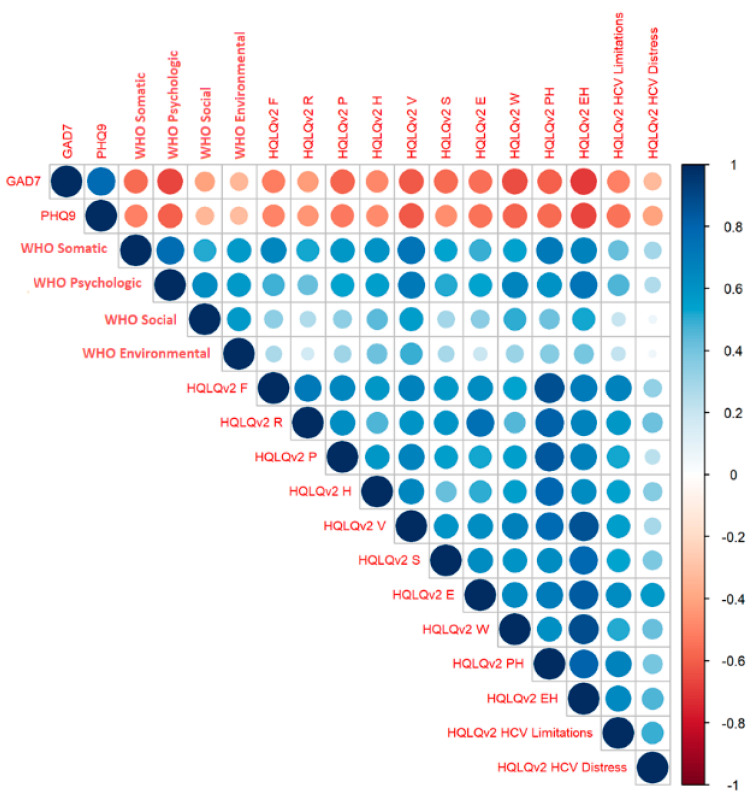
Spearman’s Rank correlations for all of the domains in EOT. EOT—end of the treatment; HQLQ v.2—Hepatitis Quality of Life Questionnaire; F—Physical Functioning; R—Role Physical; P—Bodily Pain; H—General Health; V—Vitality; S—Social Functioning; E—Role Emotional; W—Wellbeing; PCS—Physical Component Score; MCS—Mental Component Score; GAD7—Generalized Anxiety Disorder; PHQ9—Patient Health Questionnaire.

To assess the relationship between the domains in different questionnaires, Spearman’s Rank correlation was performed (Figure 5).

Decreased scores on the psychological scale were clearly correlated with worsening anxiety and depression in the GAD-7 and PHQ-9. Elevated scores for depression and anxiety impaired most aspects of quality of life and were clearly associated with decreased mental health (MCS), well-being (W), and vitality (V), but were also predictive of poorer physical health (PH). The association between the PHQ-9 and GAD-7 was also found to be relevant, reflecting the co-existence of anxiety with depression.

The weakest association with the other subscales was presented by the environmental domain of the WHOQOL-BREF questionnaire. However, higher scores were most significantly related with better vitality (V) and well-being (W) in the HQLQ v.2. The WHOQOL-BREF’ social domain was also weakly correlated with the other subscales, but increased scores on this subscale were related with improved well-being on the HQLQ v.2.

## 4. Discussion

Current knowledge suggests that interferon- and ribavirin-free DAA therapies have less impact on patients’ quality of life. Many papers were published on this issue in recent years, but most are based on data from clinical trials. According to this knowledge, DAA regimens are associated with minimal impairment of quality of life and rapid recovery from treatment, especially in those achieving SVR [23,24]. Due to this fact, noticing even discrete changes in quality of life became possible.

HCV-related depression and fatigue are one of the important factors negatively affecting quality of life. Depression is reported to be 1.5 to 4 times more common among HCV-infected patients compared to the population average [5].

In the studied population, depression and anxiety rates (35.2% and 30%) at the baseline were twice as high as the average for the general Polish population [25]. These observations are consistent with reported by other investigators. Adinolfi et al. [26] reported the presence of depression and/or anxiety among 1/3 of HCV-infected patients. The major depressive disorder and generalized anxiety were found in 5.8–7% of patients. In another study [27], the proportion of patients with major depression at the baseline ranged in different arms from 5.2–8.3%. These observations are consistent with ours, where 6.8% of patients showed features of severe depression and 13.5% showed features of severe anxiety, which is 10 times higher than in the general population (0.7%) [25].

Comorbidity of anxiety and depression can impact socioeconomic outcomes, affecting education, job performance, and, indirectly, treatment effectiveness. Blackwell et al. [28], in a large analysis of health care system data, found that the presence of psychiatric disorders reduces the linkage-to care and increases the risk of treatment discontinuation. Depression is even more common among patients with cirrhosis. Barboza et al. [29], in a study of 43 patients with F4 cirrhosis, found the presence of depressive disorders in almost 70% of patients, and identified that a 1-point increase in quality of life results in a 4% decrease in mortality.

Likewise, we observed the effect of depression and anxiety on the deterioration of quality of life in the WHOQOL-BREF questionnaire. Scoring >10 on the GAD-7 and PHQ-9 was associated with a significant decrease in scores across all of the domains (Table 4).

We demonstrated a positive effect of DAA treatment on anxiety and depression symptoms among patients with HCV, however, the effect of different regimens was not analyzed. The percentage of patients who scored >10 points on the PHQ-9 at baseline decreased to 18.2%. The number of patients with symptoms of severe depression also decreased to 1% after treatment. A total of 83.3% of patients with the most severe symptoms changed their depression severity to a lower classification. However, this result was not statistically significant, probably due to the small number of patients in this group. The significant change in the classification was present in patients with less severe depression symptoms. In terms of perceived anxiety, we observed a similar trend: the percentage of patients with >10 points on the GAD-7 decreased to 19.1%, and those with severe anxiety from 13.5% to 5.6%. The effect of DAA treatment on anxiety was significant in all of the groups, except patients with lower fibrosis and those previously treated with INF. 

Similar real life analysis, comparing the impact of different DAA treatment regimens on depressive symptoms among patients with hepatitis, was published in 2018 [27]. The successful treatment was associated with improvement in perceived depression, while DAA therapy, when extended to 24 weeks, resulted in a temporary worsening.

Likewise, Nardelli et al. [30] showed that effective DAA treatment leads to improvement in 7/10 subscales of the SF-36 except for R, P, and S. The values after the treatment were similar to those obtained in a healthy population. These results are consistent with ours, where we observed statistically significant differences in 8/10 SF-36 subscales, except for R and F. However, our results were lower than those obtained in the Italian cohort. One reason could be that the patients in our study were not aware of the treatment results at EOT, and we were able to avoid the psychological boosting effect. [17].

In our study, improvement in the HRQoL in the WHOQOL-BREF general questionnaire was significant. The values obtained after treatment in the psychological domain were even higher than the mean values for the healthy population (74.3 vs. 70.6). In other domains, the post-treatment values were statistically higher than baseline, but did not reach the values observed in the population norms. This effect was reported by other investigators [15], but at extended follow-up, the values reached population norms.

We observed that patients with more advanced fibrosis scored worse in anxiety and depression at baseline. In addition, depression and anxiety were determinants of reduced HRQoL, especially in the psychological and social domains. This observation is in line with Juanbeltz et al. [31], where the strongest factor for reduced quality of life was baseline severe depression, anxiety, and advanced fibrosis. At the same time, patients with the lowest baseline scores received a major benefit from successful DAA treatment.

We demonstrated that HCV eradication had a significant impact on psychological and environmental domain in the WHOQOL-BREF questionnaire among female but not male, respondents. Huang et al. [32] describe that female gender is a significant factor for reduced HRQoL. In our study, male gender was a predictor of higher quality of life in the social domain. This observation may be due to the fact that HCV has a greater effect on perceived anxiety and depression among female respondents, as illustrated by the lower baseline scores on the PHQ-9 and GAD-7.

Health surveys are used in clinical practice in many countries to improve the quality of health care. Examples include the Swedish Rheumatology Quality Registry, the HowsYourHealth.org patient portal in the US primary care model, or the Dutch FeedForward application for among children with cancer. Nelson et al. [33] provide evidence that the use of quality-of-life questionnaires in clinical practice leads to better physician–patient communication, helps tailor treatment plans to patient preferences and needs, and improves patient satisfaction with health care. Decreased levels of depression and anxiety and improved quality of life was observed among patients who were enrolled in a nurse-led health education program [34].

The limitation of this study was a small sample size, with reduced possibility of assessing the differences among subpopulations, as a single center study. Nonetheless, the characteristics of patients treated for HCV in Poland, based on the data published by Robert Flisiak et al. from the same period [35] by the distribution of age, gender, and fibrosis stage, were comparable to our data.

Considering the lack of information on treatment regimens in individuals, an analysis on the effect of particular therapies was not possible.

The results presented in our study are illustrating the importance of health perception among chronically ill patients. Anti-HCV screening campaigns dedicated to patients receiving care from psychiatrists and psychologists could potentially result in new HCV diagnoses. Education of psychological clinic staff and awareness-raising among psychiatrists could also help increase HCV detection.

## 5. Conclusions

High anxiety and depression prevalence in chronic hepatitis C, involving approximately one in three of the HCV-infected, is the main driver of decreased health-related quality of life. The significant improvement in anxiety and depression after HCV eradication supports anti-HCV screening campaigns among patients in psychiatric and psychological care.

Introduction of mandatory PHQ-9 and GAD-7 screening tests in HCV-infected would help to identify patients with worse adherence and risk of suicide and implement psychiatric treatment.

## Figures and Tables

**Table 1:** Demographic characteristic, *n* = 90.

Variable	Category	Total	Missing Data, *n* (%)
Age (years) (mean ± SD)		43.8 ± 12.6	0 (0.0%)
Sex, *n* (%)	Female	45 (50.0)	0 (0.0%)
	Male	45 (50.0)	0 (0.0%)
Time since diagnosis-categories (years), *n* (%)	0–2	34 (38.2)	1 (1.1%)
	3–4	20 (22.5)	
	5–10	12 (13.5)	
	>10	23 (25.8)	
Fibrosis-categories, *n* (%)	F0	3 (3.3)	0 (0.0%)
	F0/F1	6 (6.7)	
	F1	25 (27.8)	
	F1/F2	2 (2.2)	
	F2	21 (23.3)	
	F2/F3	5 (5.6)	
	F3	19 (21.1)	
	F4	9 (10.0)	
Fibrosis-categories, *n* (%)	F0–F2	57 (63.3)	0 (0.0%)
	F3–F4	33 (36.7)	
Earlier HCV treatment, *n* (%)	No	75 (84.3)	1 (1.1%)
	Yes—interferon	14 (15.7)	

HCV—hepatitis quality of life.

**Table 2:** GAD-7, PHQ-9 summary score at the baseline and at EOT.

Variable	Baseline Summary Score	EOT Summary Score	Missing Data, *n* (%)	*p*
**GAD-7, *n***	89	89	1 (1.1%)	<0.001
mean ± SD	7.6 ± 5.1	5.6 ± 4.9		
median [Q1–Q3]	6.0 [4.0, 12.0]	5.0 [1.0, 8.0]		
(min–max)	0.0–20.0	0.0–21.0		
**PHQ-9, *n***	88	88	2 (2.2%)	<0.001
mean ± SD	8.2 ± 6.4	5.5 ± 5.2		
median [Q1–Q3]	7.0 [3.0, 11.0]	4.0 [1.0, 8.0]		
(min–max)	0.0–25.0	0.0–23.0		

EOT—end of the treatment; GAD-7—Generalized Anxiety Disorder Questionnaire; PHQ-9—Patient Health Questionnaire; SD—standard deviation.

**Table 3:** Th results of WHOQOL-BREF Questionnaire transformed 0–100 scores at the baseline and at EOT.

WHOQOL-BREF Domain		Baseline, *n* = 90	EOT, *n* = 90	*p*
**somatic**	median [Q1–Q3]	60.7 [50.0, 74.1]	71.4 [60.7, 81.2]	<0.001
**psychologic**	median [Q1–Q3]	70.8 [62.5, 79.2]	75.0 [66.7, 83.3]	0.004
**social**	median [Q1–Q3]	75.0 [58.3, 81.2]	75.0 [66.7, 75.0]	0.186
**environmental**	median [Q1–Q3]	65.6 [59.4, 74.2]	71.9 [62.5, 78.1]	0.003

WHOQOL-BREF—The World Health Organization Quality of Life—BREF; EOT—end of the treatment.

**Table 4:** The results of HQLQ v.2 Questionnaire normalized scores at baseline and at EOT.

HQLQv.2 Domain	Baseline, Mean ± SD *n* = 90	EOT, Mean ± SD *n* = 90	*p*
F	58.81 ± 6.61	59.71 ± 5.84	0.189
R	58.36 ± 8.36	59.67 ± 6.87	0.129
P	52.83 ± 9.02	55.22 ± 9.63	0.031
H	52.60 ± 9.07	55.55 ± 8.15	<0.001
V	53.56 ± 14.09	58.63 ± 13.06	0.001
S	63.45 ± 16.58	68.87 ± 15.03	0.004
E	55.02 ± 8.37	57.13 ± 6.80	0.005
W	55.78 ± 11.84	58.95 ± 11.42	0.014
PCS	55.98 ± 6.36	57.32 ± 6.42	0.037
MCS	56.06 ± 13.27	60.53 ± 11.28	0.005
HCV Limitations	58.42 ± 12.61	63.10 ± 11.96	<0.001
HCV Distress	79.62 ± 22.84	84.30 ± 20.46	0.034

HQLQ v.2—Hepatitis Quality of Life Questionnaire; F—Physical Functioning; R—Role Physical; P—Bodily Pain; H—General Health; V—Vitality; S—Social Functioning; E—Role Emotional; W—Wellbeing; PCS—Physical Component Score; MCS—Mental Component Score; SD—standard deviation; EOT—end of the treatment.

**Table 5:** The effect of depression on lowering the scores of the WHO BREF scales.

**WHOQOL-BREF Domain**	**No Anxiety (<10)** **Mean ± SD**	**Anxiety (>10)** **Mean ± SD**	***p***
somatic	22.50 ± 2.77	19.00 (3.15)	<0.0001 ^2^
psychologic	22.19 ± 2.40	19.89 (2.83)	0.0062 ^1^
social	11.71 ± 1.93	9.96 (2.71)	0.0008 ^1^
environmental	29.90 ± 3.88	27.22 (3.49)	0.0029 ^2^
**WHOQOL-BREF Domain**	**No Depression (<10)** **Mean ± SD**	**Depression (>10)** **Mean ± SD**	***p***
somatic	22.82 ± 2.68	19.03 ± 3.02	<0.001 ^1^
psychologic	22.26 ± 2.42	20.03 ± 2.80	<0.001 ^1^
social	11.81 ± 1.88	9.97 ± 2.64	<0.000 ^2^
environmental	30.00 ± 3.94	27.58 ± 3.58	0.004 ^1^

^1^ Mann–Whitney-U; ^2^ Student’s *t*-test; WHOQOL-BREF—The World Health Organization Quality of Life-BREF; SD—standard deviation.

## Data Availability

Not applicable.
